# Impact of introduction of Xpert MTB/RIF test on tuberculosis (TB) diagnosis in a city with high TB incidence in Brazil

**DOI:** 10.1371/journal.pone.0193988

**Published:** 2018-03-08

**Authors:** Giovana Rodrigues Pereira, Márcia Silva Barbosa, Natan José Dutra Dias, Carlos Podalirio Borges de Almeida, Denise Rossato Silva

**Affiliations:** 1 Programa de Pós-Graduação em Ciências Pneumológicas da Universidade Federal do Rio Grande do Sul, Porto Alegre, Brazil; 2 Setor de Tuberculose, Laboratório Municipal de Alvorada, Alvorada, Brazil; 3 Microbiologia, Faculdade Factum, Porto Alegre, Brazil; 4 Universidade Federal de Ciências da Saúde de Porto Alegre, Porto Alegre, Brazil; 5 Faculdade de Medicina, Universidade Federal do Rio Grande do Sul, Porto Alegre, Brazil; Indian Institute of Technology Delhi, INDIA

## Abstract

**Background:**

Xpert MTB/RIF is increasingly used in many countries as the initial diagnostic test for tuberculosis (TB). Few studies have evaluated the effect of Xpert on TB diagnosis under programmatic conditions in Brazil. The aim of the present study was to evaluate the impact of introduction of Xpert MTB/RIF on TB diagnosis in a city with high TB incidence in Brazil.

**Methods:**

We included patients evaluated with conventional diagnostic tests during one year before Xpert introduction (pre-Xpert group) and patients evaluated using Xpert during one year after the test introduction (post-Xpert group).

**Results:**

620 patients met the inclusion criteria (208 in the pre-Xpert group and 412 in the post-Xpert group) and were included in the analysis. The time until TB diagnosis was shorter in post-Xpert group (0.7 day, IQR: 0.5–1.0 day) than in pre-Xpert group (2.0 days, IQR: 2.0–2.0 days) (p<0.0001). Atypical disease characteristics, such as less weight loss, fever, dyspnea, night sweats, and hemoptysis; a negative sputum smear; a negative culture, and a chest X-ray atypical of TB were more common in post-Xpert group than in pre-Xpert group (p<0.0001 for all).

**Conclusions:**

We found that the implementation of the Xpert MTB/RIF assay, under programmatic conditions, improve and facilitate TB diagnosis, especially in cases with atypical disease manifestations. These results are likely to be generalizable to settings with a similar high TB incidence.

## Introduction

Across the world tuberculosis (TB) remains an important public health problem, especially in developing countries. It is estimated that one third of the world population is infected with Mycobacterium tuberculosis. Brazil is in 18th place among the 22 countries that collectively account for 80% of TB cases globally, with reported incidence of 32.4 cases/100,000 inhabitants/year in 2016 [1, 2].

World Health Organization (WHO) recommends smear microscopy as the initial diagnostic test for TB. However, more than 40% of new pulmonary TB cases were smear-negative [2]. Sputum culture for mycobacteria has greater sensitivity in comparison with smear microscopy. Nevertheless, culture can take up to 8 weeks to get results, being less useful in clinical practice [3].

Xpert MTB/RIF assay has been introduced for the diagnosis of TB and rifampicin resistance in 2010. The assay can be performed directly from a clinical sputum sample or from a decontaminated sputum pellet and can generally be completed in less than two hours. Xpert is increasingly used in many countries as the initial diagnostic test for TB [4, 5]. Few studies have evaluated the effect of Xpert on TB diagnosis under programmatic conditions in Brazil [6, 7]. Therefore, the aim of the present study was to evaluate the impact of introduction of Xpert MTB/RIF on TB diagnosis in a city with high TB incidence in Brazil.

## Materials and methods

### Study design and location

We conducted a cross-sectional study in an outpatient TB clinic in Alvorada, RS, Brazil. Alvorada is a city in the metropolitan area of Porto Alegre, which is the fourth Brazilian capital with the highest number of TB cases, with an incidence of 80.4 cases/100,000 inhabitants [1]. The study was approved by the Ethics Committee of Hospital de Clínicas de Porto Alegre in January 15^th^, 2016 (number 16–0063).

The present study was designed to investigate the impact of Xpert introduction. We included patients evaluated with conventional diagnostic tests during one year (March 2014-March 2015) before Xpert introduction (pre-Xpert group) and patients evaluated using Xpert during one year (May 2015-May 2016) after the test introduction (post-Xpert group). We did not include patients evaluated during one month (April 2015) between these two periods, as it was a transition period, when Xpert was rolled out in Brazil. Smear microscopy was not a pre-requisite for Xpert; the test was performed in both smear positive and negative cases.

### Patients

Outpatients aged > 18 years with respiratory symptoms suggestive of pulmonary TB, like productive cough for > 2 weeks, cough of any duration accompanied by constitutional symptoms (fever for at least 3 days, night sweats or weight loss of at least 3 kg in the previous month), or hemoptysis, who were able to collect a sputum sample were included in the study. HIV positive and smear-negative pulmonary TB cases were included. Patients with extrapulmonary TB and those who were unable to collect a sputum sample were excluded from this study. Pulmonary TB was diagnosed according to the Brazilian Guidelines for Tuberculosis [3].

### Data collection

Demographic data and medical history were collected from patient records using a standardized data extraction tool, described in detail previously [8, 9]. Chest X-rays (CXRs) were classified as typical of TB or compatible with TB, according to previously described guidelines [10].

Sputum smears were stained by Ziehl-Neelsen (ZN) staining technique for the detection of AFB, and culture was performed using the Ogawa-Kudoh method. The Xpert MTB/RIF test was performed according to manufacturer’s instructions (Cepheid, Sunnyvale, California, USA, 2013).

### Statistical analysis

Data analysis was performed using SPSS 18.0 (Statistical Package for the Social Sciences, Chicago, Illinois) and MedCalc 16.4.3 software package (MedCalc Software, Mariakerke, Belgium). Data were presented as number of cases, mean ± standard deviation (SD), or median with interquartile range (IQR). Categorical comparisons were performed by chi-square test using Yates’s correction if indicated or by Fisher’s exact test. Continuous variables were compared using the *t*-test or Wilcoxon test. A two-sided p value < 0.05 was considered significant for all analyses.

Positive mycobacterial culture results were defined as a gold standard of the diagnosis. On the basis of culture results, we calculated the sensitivity, specificity, positive predictive value, and negative predictive value with 95% CIs of the Xpert MTB/RIF for the detection of TB. We constructed receiver operating characteristic (ROC) curves for Xpert MTB/RIF.

In order to calculate the sample size, it was considered that the sensitivity of Xpert MTB/RIF varies from 70% to 98% [4], depending on the smears results. Thus, with a confidence interval of 95% and a power of 80%, it will be necessary to include at least 246 patients to calculate sensitivity, specificity, positive predictive value, and negative predictive value. For the comparison of the pre- and post-Xpert groups, considering an alpha error of 0.05 and a beta error of 0.20, 121 patients will be required per group [11].

## Results

During the study period, 620 patients met the inclusion criteria (208 in the pre-Xpert group and 412 in the post-Xpert group) and were included in the analysis. The characteristics of the study population are shown in Table 1.

**Table 1:** Characteristics of study patients.

Characteristics	Pre-Xpert n = 208*	Post-Xpert n = 412*	p value
**Demographic characteristics**			
Age, yr	42.0 ± 15.9	51.5 ± 14.9	< 0.0001
Male sex	125 (60.1)	240 (58.3)	0.723
White race	148 (71.2)	318 (77.2)	0.123
**Symptoms**			
Cough	191 (91.8)	402 (97.6)	0.002
Weight loss	149 (71.6)	215 (52.2)	< 0.0001
Dyspnea	72 (34.6)	57 (13.8)	< 0.0001
Fever	110 (52.9)	72 (17.5)	< 0.0001
Night sweats	115 (55.3)	104 (25.2)	< 0.0001
Hemoptysis	28 (13.5)	12 (2.9)	< 0.0001
**Duration of symptoms, days**	60.0 (30.0–90.0)	35.0 (30.0–60.0)	< 0.0001
**HIV positive**	46 (22.1)	47 (11.4)	0.001
**Radiographic patterns**			
Typical of TB	172 (82.7)	97 (23.5)	< 0.0001
Compatible with TB	30 (14.4)	57 (13.8)	0.939
**AFB smear positive**	175 (84.1)	91 (22.1)	< 0.0001
**Culture positive**	173 (85.2)	78 (18.9)	< 0.0001
**Xpert MTB/RIF positive (detected)**	-	111 (26.9)	-
**RIF resistance**	-	4 (3.6)	-
**Time until TB diagnosis, days**	2.0 (2.0–2.0)	0.7 (0.5–1.0)	< 0.0001
**Treatment outcomes**			
Cure	145 (70.4)	116 (71.2)	0.962
Default	42 (20.4)	28 (17.2)	0.517
Death	12 (5.8)	7 (4.3)	0.672

*Data are presented as mean ± SD, n/N (%): number of cases with characteristic/total number of cases (percentage), or median (interquartile range).HIV: human immunodeficiency virus; TB: tuberculosis; AFB: acid-fast bacilli; MTB: Mycobacterium tuberculosis. RIF: rifampicin.

Cough was more frequent in post-Xpert group (n = 402, 97.6%) than in pre-Xpert group (n = 192, 91.8%) (p = 0.002). All other symptoms (weight loss, fever, dyspnea, night sweats, and hemoptysis) were more frequent in pre-Xpert group in comparison with post-Xpert group (p<0.0001 for all). The median duration of symptoms was higher in pre-Xpert group (60.0 days, IQR: 30.0–90.0 days) as compared with post-Xpert group (35.0 days, IQR: 30.0–60.0 days) (p<0.0001).The number of HIV positive patients was statistically higher in pre-Xpert group (n = 46, 22.1%) than in post-Xpert group (n = 47, 11.4%) (p = 0.001). Regarding diagnostic tests, a positive sputum smear, a positive culture, and a chest X-ray typical of TB were more common in pre-Xpert group than in post-Xpert group (p<0.0001). In addition, the time until TB diagnosis was shorter in post-Xpert group (0.7 day, IQR: 0.5–1.0 day) than in pre-Xpert group (2.0 days, IQR: 2.0–2.0 days) (p<0.0001).

**Fig 1:**
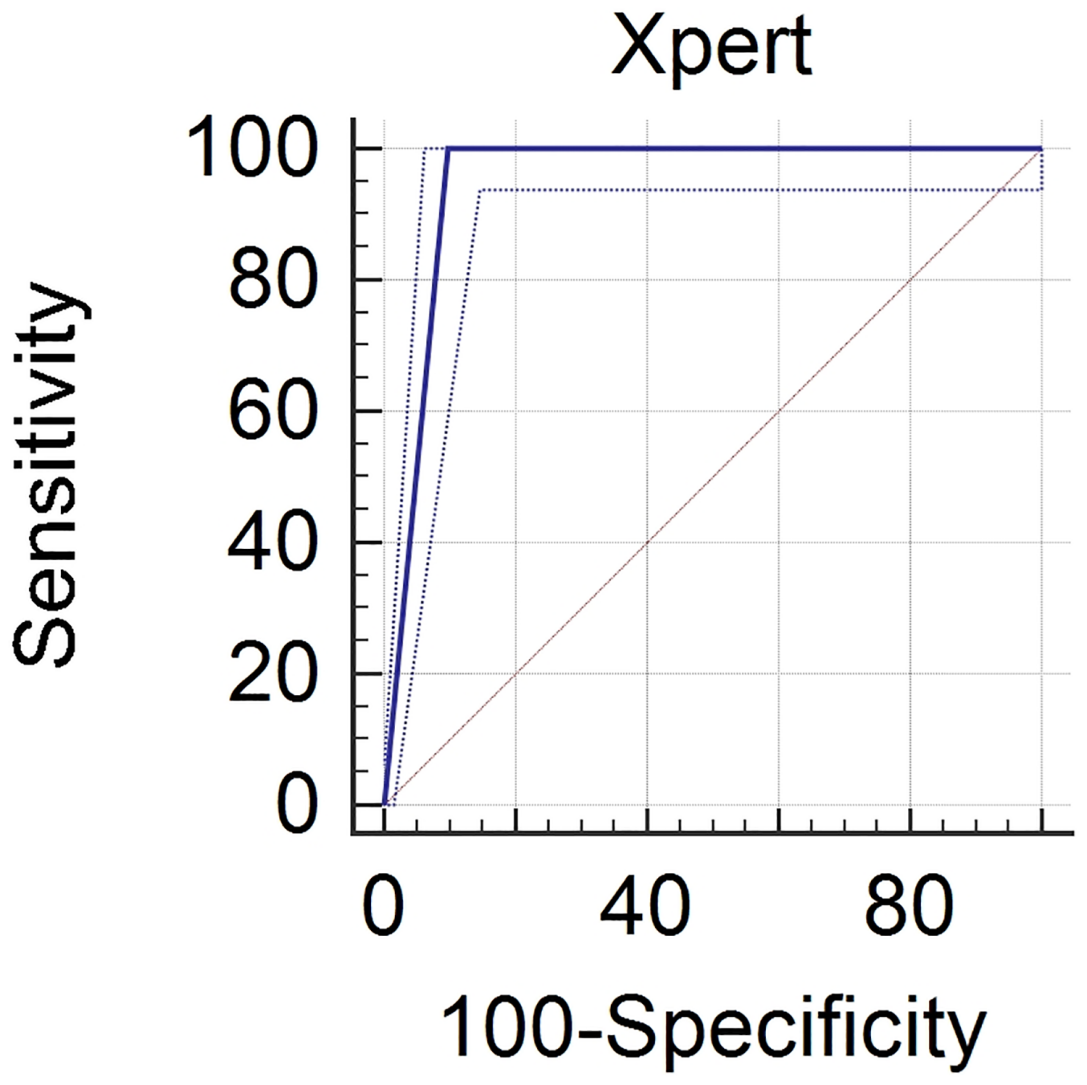
Receiver operating characteristic (ROC) curve for the detection of tuberculosis with Xpert MTB/RIF test. (The area under the ROC curve was 0.95 for the Xpert MTB/RIF test [95% CI 0.93 to 0.97; p<0.0001]).

Xpert MTB/RIF test was positive in 111/412 patients (26.9%), and RIF resistance was detected in 4/111 (3.6%) patients. Considering culture as the gold standard, the sensitivity, specificity, positive predictive value, and negative predictive value of Xpert MTB/RIF were 100.0% (95% CI 95.4–100.0), 90.1% (95% CI 86.4–93.1), 70.3% (95% CI 63.1–76.6), and 100.0% (95% CI 96.1–100.0), respectively. The area under the ROC curve was 0.95 for the Xpert MTB/RIF test (95% CI 0.93 to 0.97; p<0.0001) (Fig 1, Table 2).

**Table 2:** Sensitivity, specificity, positive and negative predictive values of Xpert MTB/RIF, using culture as gold standard.

	Culture n (%)		
Xpert MTB/RIF n (%)	Positive	Negative	Total
Positive	78 (70.3)	33 (29.7)	111
Negative	0 (0)	301 (100)	301
Total	78	334	412
	**% (95% CI)**		
Sensitivity	100.0% (95.4–100.0)		
Specificity	90.1% (86.4–93.1)		
Positive predictive value	70.3% (63.1–76.6)		
Negative predictive value	100.0% (96.1–100.0)		

CI: Confidence Interval

## Discussion

In the present study, we demonstrated that the characteristics of the patients evaluated after the introduction of Xpert test were statistically different from those evaluated pre-Xpert. They had more cough, and less frequently other symptoms, and the duration of those symptoms before diagnosis was lower. Also, a positive sputum smear, a positive culture, and a chest X-ray typical of TB were more common in pre-Xpert group than in post-Xpert group. In addition, the time until TB diagnosis was shorter in post-Xpert group than in pre-Xpert group.

The Xpert MTB/RIF test is a useful tool for early diagnosis of pulmonary TB. WHO’s current policies and guidance recommend that Xpert MTB/RIF be used as an initial diagnostic test in individuals suspected of having MDR-TB or HIV-associated TB [5]. In a study that compared four diagnostic strategies for TB, the Xpert-for-all strategy resulted in the greatest increase of TB case detection [12]. Several studies have identified Xpert MTB/RIF as a highly sensitive and specific test on both pulmonary and extrapulmonary TB [4, 13–15]. In our study, we demonstrated a sensitivity of 100% and a specificity of 90.1%. At least two previous studies also showed a sensitivity of 100% [16, 17], and Rachow A. et al [18] found a similar specificity (90.9%).

In our study, Xpert MTB/RIF established a diagnosis in a significant proportion of patients with smear-negative pulmonary TB (SNPT), and detected many TB cases missed by culture. In Brazil, up to 30% of cases of pulmonary TB are SNPT [19], but this percentage can reach 60% of all TB cases in other settings [20]. These patients have a high mortality rate, probably related to delayed diagnosis [21, 22]. We found that Xpert contributed to an earlier diagnosis, once patients were diagnosed with lower duration of symptoms in post-Xpert group than in pre-Xpert group. Additionally, the time until TB diagnosis was shorter in post-Xpert group as compared with pre-Xpert group, as demonstrated by a previous study [23]. However, the difference in the percentage of bacteriologically positive patients between pre- and post-Xpert groups should be carefully interpreted, and needs to be further studied in prospective investigations.

Although the frequency of cough was higher in post-Xpert group, other symptoms like weight loss, dyspnea, fever, night sweats, and hemoptysis were less frequent in the group evaluated after the introduction of Xpert. Respiratory symptoms and systemic manifestations are usually mild or even absent in patients with SNPT [24]. Studies have reported a decreased proportion of patients with dyspnea among SNPT patients [8, 25]. Hemoptysis is also less common in these patients [8].

SNPT is more common among HIV positive patients than in HIV negative ones [21, 26–28]. However, despite the high proportion of SNPT evaluated after Xpert introduction in the present study, the number of HIV positive patients was lower in post-Xpert group (11.4%) in comparison with pre-Xpert group (22.1%). Some previous investigations [29, 30] found no association between HIV infection and SNPT. In one of these studies [29], also conducted in Brazil, including patients with clinical-radiological suspicion of SNPT, HIV infection was not among the variables significantly associated with a diagnosis of SNPT.

Chest X-rays typical of TB were less frequent in post-Xpert group than in pre-Xpert group. It is well known that atypical chest X-ray patterns or even normal findings were more frequent in SNPT [31, 32]. Typical chest X-rays, with cavitary lesions and upper lobe involvement are less often observed in SNPT patients [24]. In this context, diagnosis of TB is therefore more challenging, and Xpert has an important role [33].

Cure rates were higher among post-Xpert group patients. Other treatment outcomes, like default and death rates were lower upon implementation of Xpert MTB/RIF; however, all these differences were not statistically significant. It was previously demonstrated that Xpert resulted in same day treatment initiation, but had no impact on tuberculosis treatment outcomes or mortality [34]. Moreover, cure rate remains below the WHO’s target of 85%, and default rates are still substantially high.

One of the limitations of this study is that we recruited patients from a single outpatient TB clinic. Furthermore, the present study did not allow us to identify differences related to costs between the periods before and after the introduction of Xpert MTB/RIF. In spite of these concerns, the knowledge of the impact of Xpert in important outcomes, such as time to treatment initiation, is relevant to TB control.

In conclusion, we found that the implementation of the Xpert MTB/RIF assay, under programmatic conditions, improve and facilitate TB diagnosis, especially in cases with atypical disease manifestations. These results are likely to be generalizable to settings with a similar high TB incidence.
